# Efficacy of 2,4-Dinitrobenzenesulfonic Acid (DNBS) in the Maintenance of a Model of Inflammatory Bowel Disease in Pigs (*Sus scrofa domestica*)

**DOI:** 10.3390/ijms26189115

**Published:** 2025-09-18

**Authors:** Dominika Szkopek, Jarosław Woliński, Łukasz Kopiasz, Katarzyna Dziendzikowska, Kamil Zaworski, Rafał Sapierzyński, Joanna Gromadzka-Ostrowska

**Affiliations:** 1 Laboratory of Large Animal Models, The Kielanowski Institute of Animal Physiology and Nutrition, Polish Academy of Sciences, Instytucka 3, 05-110 Jabłonna, Poland; d.szkopek@ifzz.pl; 2 Department of Dietetics, Institute of Human Nutrition Sciences, Warsaw University of Life Sciences, Nowoursynowska 159c, 02-776 Warsaw, Poland; katarzyna_dziendzikowska@sggw.edu.pl (K.D.); joanna_gromadzka-ostrowska@sggw.edu.pl (J.G.-O.); 3 Department of Animal Physiology, The Kielanowski Institute of Animal Physiology and Nutrition, Polish Academy of Sciences, Instytucka 3, 05-110 Jabłonna, Poland; k.zaworski@ifzz.pl; 4 Department of Pathology and Veterinary Diagnostics, Institute of Veterinary Medicine, Warsaw University of Life Sciences, Nowoursynowska 159, 02-776 Warsaw, Poland; rafal_sapierzynski@sggw.edu.pl

**Keywords:** porcine colitis model, inflammatory bowel disease, Crohn’s disease, ulcerative colitis, pigs, DNBS

## Abstract

Inflammatory bowel diseases (IBD), such as Crohn’s disease (CD) and ulcerative colitis (UC), are chronic, progressive conditions with increasing prevalence worldwide. The aim of this study was to evaluate the usefulness of a porcine model of colitis induced by 2,4-dinitrobenzenesulfonic acid (DNBS) as a translational model of IBD. Sixteen Polish White pigs were divided into a control group and colitis group. Colitis was induced by rectal administration of DNBS (80 mg/kg in 50% ethanol). Clinical status, hematological and biochemical parameters, fecal calprotectin levels, cytokine plasma concentrations, and histopathological changes in the gastrointestinal tract were evaluated. DNBS administration resulted in persistent diarrhea and mild abdominal pain without general deterioration of health. Significant increases in fecal calprotectin levels and aspartate aminotransferase (AST) activity were observed. Histopathological changes in the colon were limited to the mucosa, which is similar to human UC, while the mild changes observed in the ileum indicate similarity to CD. This model is characterized by moderate inflammation, high reproducibility, and low mortality, making it valuable model in translational research on IBD.

## 1. Introduction

Chronic inflammatory bowel disease (IBD) represents a significant challenge for contemporary medicine. More than 6.8 million people worldwide suffer from IBD, with the highest and steadily rising prevalence observed in Europe and North America [1,2,3]. The growing incidence of IBD is due, among other factors, to increasing urbanization and changes in people’s dietary patterns [4]. Given its chronic, progressive nature, the high cost of treatment, and its inconvenience to the patient, the rise in incidence of IBD implies an increasing socioeconomic burden [5,6].

Crohn’s disease (CD) and ulcerative colitis (UC) are two main types of IBD. Both are diseases of complex etiology that involve interactions between genetic predisposition, environmental factors, abnormalities in the innate and adaptive immune response, and changes in the gut microbiome [7,8]. Importantly, the specific mechanism leading to immune imbalance in CD remains unclear. Both diseases are progressive and, without timely and effective treatment, may result in irreversible and long-term complications [9], including malabsorption, malnutrition, weight loss, diarrhea, and an increased risk of colorectal cancer [10,11]. The two diseases differ mainly in their location in the gastrointestinal tract (GIT) and the extent of inflammation in the gastrointestinal wall. In UC, inflammation is limited to the mucosa and submucosa of the large intestine [12,13]. In contrast, CD is characterized by wall-to-wall transmural inflammation that can affect any part of the GIT, commencing from the mouth to conclude in the anus. Nevertheless, in 50% of cases, regardless of gender, the colon is involved [12,13]. CD is also characterized by alternating periods of exacerbation and remission. Several treatments are currently being used to alleviate clinical symptoms and manage complications of IBD; however, they often do not result in a cure and may cause significant side effects [14]. In addition, recent research has increasingly focused on microbiome-targeted therapies, including probiotics and postbiotics, which have shown potential in modulating immune responses and preserving epithelial integrity—partly through regulating mitochondrial function [15]. Despite growing interest, the precise mechanisms underlying their therapeutic effects remain poorly understood. This underscores the critical need for well-characterized, physiologically relevant, and translational animal models that can support mechanistic investigations into IBD pathophysiology and the evaluation of emerging therapeutic strategies, including nutritional and microbiota-modulating interventions [16,17].

Research on colitis requires appropriate animal models, which are essential for understanding IBD pathophysiology, as well as testing potential therapies [18,19]. To date, several colitis models have been developed that vary in terms of induction method, disease course, and immune response. Rodents (mice and rats) are the most commonly used, while the models can be divided into three main categories: chemically induced, genetically modified, and immune cell transfer-based models [20]. 2,4-dinitrobenzenesulfonic acid (DNBS) can be singled out among chemically induced models. DNBS, as a hapten, interacts with proteins in the colon tissue, triggering the immune system and leading to the infiltration of inflammatory cells [21]. Ethanol, which is used as a solvent, disrupts the initial mucosal barrier of the colon, allowing DNBS to penetrate the lamina propria. This process resembles changes observed in patients with CD. Although DNBS has been used extensively to replicate colitis-like conditions in rodents, only a limited number of studies have evaluated its efficacy in large animal models, such as pigs [22,23,24].

The gastrointestinal anatomy and physiological similarity between humans and pigs regarding digestive and associated metabolic processes place these animals in a superior position over other non-primate models, including rodents [25]. Moreover, pigs have comparable nutritional requirements and similarities to humans in intestinal microbiota composition, metabolism, and immune responses [25,26]. For this reason, the domestic pig is regarded as an excellent biomedical animal model for the comprehension of many diseases, including CD and UC, and studies performed on these animals are widely considered preclinical research [23,26,27,28,29]. In this study, we hypothesized that the domestic pig represents a suitable research model for analyzing pathogenetic mechanisms and testing new therapeutic approaches for IBD. For this purpose, we evaluated the features of DNBS-induced colitis, with particular attention to the animals’ clinical condition, blood counts, liver aminotransferases activities, cytokines levels, body weight and weight gain, histopathological and histomorphometric changes in the colon and small intestine, and fecal calprotectin levels. Analysis of these parameters allows a comprehensive evaluation of the DNBS effect and helps determine the usefulness of this model in colitis research. Our findings may contribute to a better understanding of the mechanisms underlying the development and maintenance of gut inflammation, as well as support the development and testing of new therapies, including drugs, probiotics, or dietary interventions.

## 2. Results

### 2.1. Body Weight and Body Weight Gain

**Figure 1:**
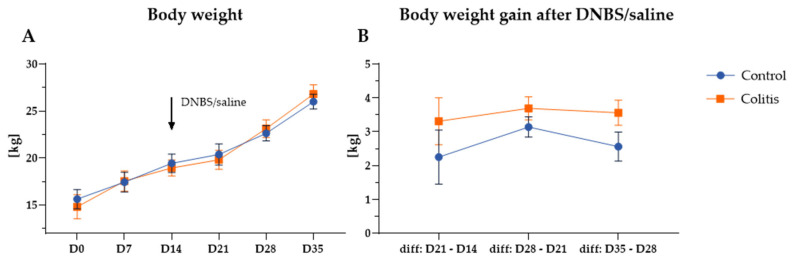
(**A**)—Pig’s body weight during the experiment. (**B**)—Pig’s body weight gain during the experimental period. Abbreviations: D0—day 0 of the experiment, before administration of saline or DNBS, D7—day 7 of the experiment, before administration of saline or DNBS, D14—day 14 of the experiment, end of adaptation period and DNBS/saline administration, D21—day 21 of the experiment, 1 week after administration of saline or DNBS, D28—day 28 of the experiment, 2 weeks after administration of saline or DNBS, D35—day 35 of the experiment, 3 weeks after administration of saline or DNBS, diff—difference. Results are presented as the mean ± SEM. No significant differences (repeated measures ANOVA, *p* > 0.05).

For the entire experimental period, there were no significant differences in body weight (BW) and body weight gain (BWG) between the control group and the colitis group (*p* > 0.05). The results of BW and BWG are shown in Figure 1.

### 2.2. Clinical Condition of Animals in the Experimental Period

In the control group, none of the pigs showed signs of abdominal pain throughout the study period (AP = 0, 8/8). In contrast, two pigs (2/8) exhibited moderate pain in the colitis group on the first day after DNBS administration (AP = 1). However, no pigs in either group showed signs of severe abdominal pain. No abdominal soreness was noted during the later stages of the study. In the control group, the quality and frequency of feces on most days were normal (FQF = 0). Transient fecal changes were observed occasionally—6/8 pigs had too soft feces (FQF = 1) and single cases of diarrhea occurred (5/8, FQF = 2). Most often, these disturbances appeared about 2–7 days after administration of 0.9% NaCl. All pigs (8/8) in the colitis group experienced diarrhea for at least several days. The most common value observed was FQF = 2. Additionally, three pigs (3/8) had episodes of diarrhea with blood (FQF = 3), especially during the first few days after DNBS administration. Symptoms at FQF = 1 or FQF = 2 persisted throughout the study period in the colitis group. None of the animals in either group showed symptoms such as decreased appetite, thirst, activity, degree of hydration, environmental interaction, and increased or decreased internal body temperature and respiratory rate. The results from the point score of the abdominal pain and the quality and frequency of feces are shown in Table 1.

### 2.3. Hematological and Biochemical Examination of Blood

**Figure 2:**
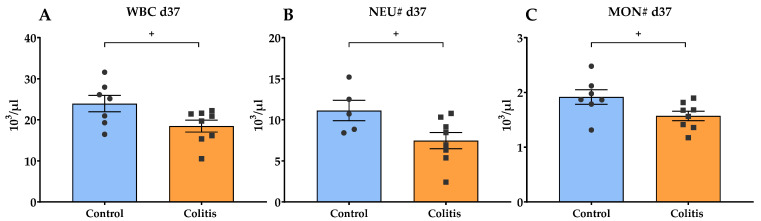
The white blood cell count (WBC, (**A**)), absolute value of neutrophils (NEU#, (**B**)), and absolute value of monocytes (MON#, (**C**)) in the peripheral blood of pigs on day 37 of the experiment. Results are presented as mean ± SEM. Significant differences are indicated by + (*p* < 0.05).

The blood morphological examination showed a significant decrease in the white blood cell count (WBC) (*p* = 0.042), absolute value of neutrophils (NEU#) (*p* = 0.041), and monocytes (MON#) (*p* = 0.044) at the end of the experiment in the colitis group. However, all values remained within the hematological norms for the species (WBC 10.20–30.00 10^3^/μL, NEU# 2.80–13.80 10^3^/μL, MON# 0.20–2.60 10^3^/μL) (Figure 2). There were no significant differences between the two groups in other hematological parameters at days 15 and 37 (*p* > 0.05) (Supplementary Table S1). The plasma activity of liver enzymes such as alanine aminotransferase (ALT) and aspartate aminotransferase (AST) is commonly used as a marker of hepatic function. While no significant differences in ALT activity were observed between groups or time points, AST activity was significantly higher in the colitis group on day 37 compared to the control group (*p* = 0.0153). Moreover, AST activity in the colitis group increased significantly between day 15 (before DNBS administration) and day 37 (*p* = 0.0080) (Table 2).

### 2.4. Plasma Cytokines Concentrations

To evaluate the systemic inflammatory response, plasma concentrations of the pro-inflammatory cytokines tumor necrosis factor alpha (TNF-α) and interleukin-1 beta (IL-1β) were measured. No statistically significant differences in the levels of either cytokine were observed between the experimental groups at any time point (Table 3).

### 2.5. Fecal Calprotectin Concentration

**Figure 3:**
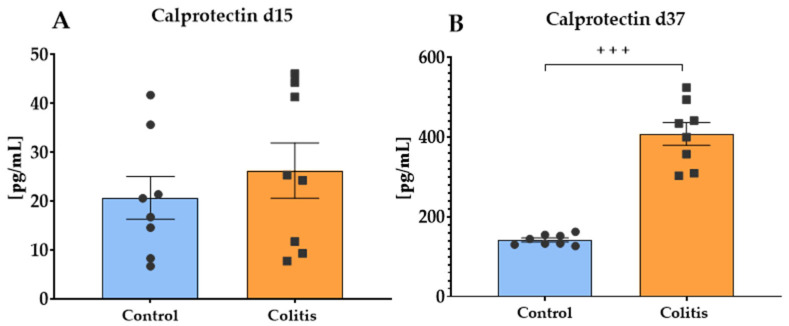
Calprotectin concentration on days 15 (**A**) and 37 (**B**) of the experiment in pig feces. Results are presented as mean ± SEM. Significant differences are indicated by +++ (*p* < 0.001).

The concentration of calprotectin in feces on day 15 of the study (Figure 3A) did not differ significantly between the control (mean 20.7 pg/mL) and the colitis (mean 26.26 pg/mL) group (*p* = 0.450). However, on day 37 (Figure 3B), calprotectin concentration was significantly higher in the colitis group (mean 407.89 pg/mL) compared to the control group (mean 140.07 pg/mL) (*p* = 0.0002).

### 2.6. Histopathological Evaluation of Porcine Colon

**Figure 4:**
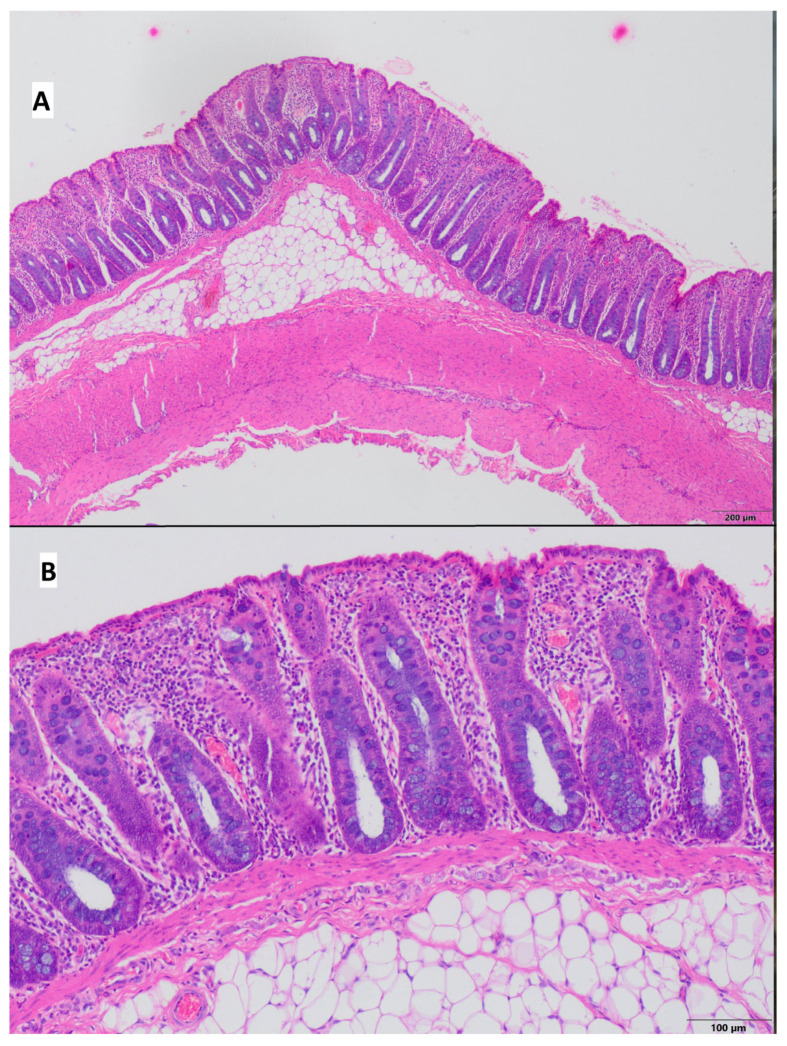
Representative microscopic view of colonic mucosa of a pig from the control group—mucosa without erosions and ulceration is visible; hematoxylin–eosin stain, (**A**)—magnification 40×, (**B**)—magnification 100×.

**Figure 5:**
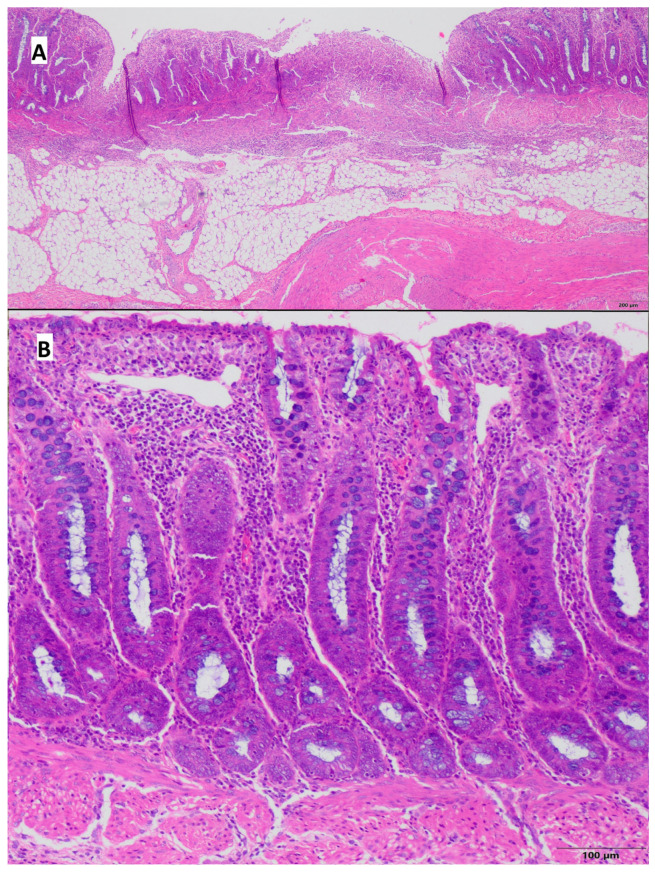
Representative microscopic view of the colonic mucosa of a pig from the colitis group. (**A**) Erosions and ulcerations of mucosa are visible; however, lesions are restricted to mucosa without involvement of submucosa and muscular layer; hematoxylin–eosin stain, magnification 40×; (**B**) Area of mucosa not affected by ulceration, diffuse inflammatory infiltrate consisting of small lymphocytes and plasma cells in lamina propria is visible; hematoxylin–eosin stain, magnification 100×.

Pathological lesions were restricted to the mucosa, with chronic inflammatory infiltrate consisting of small lymphocytes and plasma cells. In the control group, the average lesions were estimated as 1.0 according to Geboes scoring scale [30], whereas in the colitis group, it was 1.62. Neutrophils in the epithelium of intestinal crypts were present in 3 out of 8 pigs from the control group and in 5 out of 8 pigs from the colitis group; however, these cells were not present in the crypts lumen (crypt abscesses). Alterations in general architecture, including crypts distention, were observed in only 1 of 8 pigs from the control group and in 5 of 8 pigs from the colitis group. Erosions and ulcers were seen only in the colitis group (50% of animals), and they were absent in pigs from the control group. The results are shown in Table 4, Figure 4 and Figure 5. In addition, during tissue collection, macroscopic changes were visible in the colon of pigs at the site of induced inflammation (Supplementary Figure S1).

**Figure 6:**
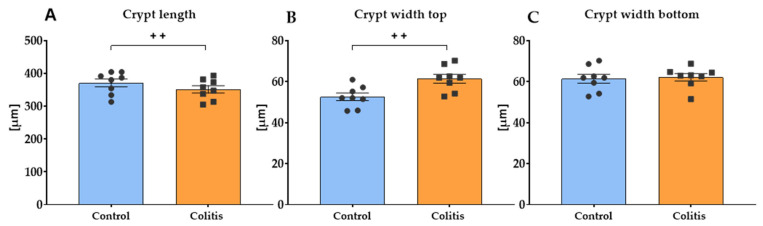
A histomorphometric analysis of the crypt length (**A**), crypt width top (**B**), and crypt width bottom (**C**) of the porcine colon. Results are presented as the mean ± SEM. Significant differences are indicated by ++ (*p* < 0.01).

A histomorphometric analysis of the colon (Figure 6) showed a significant effect of induced inflammation on the length and width of the upper part (top) of the intestinal crypts (*p* < 0.01), but no effect on the width of the intestinal crypts in the lower part (bottom). In the group with induced inflammation, shorter crypt length and higher width at the top were observed.

### 2.7. Histomorphometric Analysis of the Small Intestine

There were no significant differences in villus length, mucosa thickness, crypt depth, and muscularis thickness of the duodenum, as well as the proximal and distal parts of the jejunum, between the control and colitis group (*p* > 0.05). Significant differences were found in muscularis thickness of the middle jejunum (*p* = 0.03) and the ileum (*p* = 0.0092), and in crypt depth in the ileum (*p* = 0.0095). Other histomorphometric parameters in the ileum and middle jejunum were not significantly different between the study groups. The results are shown in Table 5. Additionally, there were also no significant differences in the length of the aforementioned intestinal segments in the two study groups (*p* > 0.05).

## 3. Discussion

The present study aimed to confirm the induction of DNBS colitis in a domestic pig model. Given the significant anatomical and physiological differences between humans and rodents [31], the use of rodent models alone has undoubtedly hindered progress and complicated the translation of research findings into effective IBD therapies [32]. In this context, the domestic pig model seems particularly valuable for translational research on IBD. Unlike mouse and rat models, pigs are more similar to humans in anatomy, physiology, body size, immunology, and genome [29,33]. This species has a single-chambered stomach and demonstrates a high degree of similarity to humans regarding inflammatory response, intestinal mucosal and muscular layer thickness, intestinal microbiota composition, nutritional requirements, and expression of immune receptors, among other features [23,25,26]. This characteristic makes this species a much more appropriate model for studying inflammatory diseases of the GIT, especially in the context of gut immunology, microbiota, or morphology. In our study, we used male domestic pigs. According to the available scientific literature, from an epidemiological point of view, Crohn’s disease occurs significantly more often in males than in females [34,35]. The use of male animals further reduces the impact of hormonal fluctuations, allowing for more homogeneous results. However, establishing separate animal models for UC and CD remains challenging due to the similar pathological changes induced by existing protocols [36,37].

In the present study, DNBS was used to induce colonic inflammation. Its mechanism of action is based on activation of the immune system through protein haptenation in the presence of 50% ethanol, which damages the intestinal barrier and allows DNBS to penetrate into the lamina propria [21]. The administration of DNBS results in the recruitment of inflammatory cells, such as neutrophils and macrophages, and increases pro-inflammatory cytokines, characteristic of the inflammatory response seen in CD. In the conducted experiment, DNBS was administered rectally in an ethanol solution at a dose of 80 mg/kg body weight (concentration 8.22% *v*/*v*), in accordance with previously described protocols [24,38]. This method is characterized by high reproducibility, the ability to modulate the severity of inflammation by adjusting the dose and frequency of application, and moderate toxicity. Another substance often used to induce colitis is 2,4,6-trinitrobenzenesulfonic acid (TNBS), which acts similarly to DNBS but has higher toxicity and mortality, as well as increased risk of tissue perforation and necrosis, especially at higher doses [39,40,41]. Antoniou et al. [41] suggest that TNBS may better mimic IBD but is more difficult to dose and may have less predictability in large animal models. Furthermore, compared to DNBS, it is considered a dangerous substance due to its strong oxidative properties, which may pose a risk of explosion when in contact with bases such as sodium and potassium hydroxide [21]. Dextran sulfate sodium (DSS) is another commonly used compound for inducing colitis, especially in rodent models. It exerts a direct toxic effect on intestinal epithelial cells, leading to necrosis and an acute inflammatory response [42]. DSS-induced colitis of an acute nature that primarily involves the mucosa and submucosa of the large intestine, without the characteristic transmurality present in CD, is therefore more similar to UC [43]. Moreover, DSS has nephrotoxic and hepatotoxic effects, which limit its use in long-term studies [44]. The above disadvantages of DSS, as well as the variability in concentration and inconsistent exposure to the agent, lead to heterogeneity in results and limit the ability to compare results between study groups [21]. In contrast, DNBS more effectively reproduces chronic and moderate disease courses with relatively low mortality [21,24,38] and may represent a compromise between the properties of DSS and TNBS in large animal models. Moreover, DNBS-induced colitis in pigs more closely replicates the characteristics of intestinal inflammation observed in human CD than DSS-based rodent models, which are generally considered to better mimic UC. It therefore presents a promising model for translational research in CD.

In this study, DNBS administration in pigs resulted in typical clinical signs of colitis, in the form of diarrhea, with occasional presence of blood in the stool and transient abdominal soreness. These symptoms occurred early after inflammation induction and were not observed in the control group. Notably, general clinical condition—including appetite, hydration status, activity, and vital signs—remained stable in all animals throughout the study, resembling the mild to moderate course of CD observed in humans [9,24]. The clinical observation of diarrhea is consistent with the findings of Zizzo et al. [38], who also reported altered stool consistency beginning the day after DNBS administration in rats. Palenca et al. [24] studied a mouse model of colitis induced by DNBS and found that it caused stable inflammation of moderate severity. The main clinical signs observed were diarrhea and changes in stool composition, while the effect on the appetite and activity of the animals was limited. These findings are consistent with our results. Despite GIT-related symptoms, no significant differences in body weight or weight gain were observed between the control and colitis groups. The study by Żyła et al. [45] showed that after TNBS administration, rats exhibited significantly reduced feed intake and body weight gain, which correlated with full clinical CD manifestations, including bloody diarrhea. Likewise, Nielsen et al. [25] observed that DSS-induced colitis in pigs resulted in a 23% reduction in feed intake and a 36% decrease in daily weight gain compared to healthy animals. Symptoms such as diarrhea, bloody stools, and weight loss may mirror the degree of gastrointestinal damage, but our results confirm that DNBS administration in a large animal model leads to mild to moderate inflammation that does not lead to cachexia over a 3-week observation period.

In the typical course of the acute inflammatory phase in IBD, patients exhibit leukocytosis and neutrophilia as a result of increased production of pro-inflammatory cytokines [26]. However, hematological analysis in the present study showed a significant reduction on day 37 in peripheral blood white blood cell (WBC) counts, as well as absolute neutrophil (NEU#) and monocyte (MON#) counts in pigs with DNBS-induced colitis. It should be noted that despite the demonstrated significance, these values were within the hematological norms for pigs. Neutrophils are the most abundant leukocyte population in the blood, comprising 50–60% of circulating leukocytes [46]. The results obtained in the present study suggest different dynamics of the inflammatory process, probably due to the stage of inflammation at week 3 after colitis induction. This interpretation is partly consistent with our previous study by Kopiasz et al. [47] in the rat model of CD, which showed that changes in the number of WBCs and their subtypes significantly depended on the time elapsed after TNBS administration. Studies on the pathogenesis of IBD have shown that, during intestinal inflammation, resident monocytes contribute to the recruitment of neutrophils through producing macrophage-derived chemokines [48,49]. One hypothesis explaining our study’s reduction in blood leukocyte levels may be their massive recruitment from the peripheral blood to the ongoing site of inflammation, as evidenced by the colon’s histopathological findings. Thus, the reduction in circulating leukocytes does not necessarily indicate an improvement in the inflammation, but rather a shift in its localization and nature. This is in line with Witko-Sarsat et al. [50] who suggested that under chronic inflammation conditions, the number of neutrophils in the peripheral blood may paradoxically decrease due to their extensive consumption in the focus of inflammation. Consistent with this interpretation, no significant differences were found in systemic concentrations of TNF-α and IL-1β between groups in the present study. Although these cytokines are considered key pro-inflammatory mediators in IBD, our findings align with other studies using TNBS or DNBS models, in which systemic levels of TNF-α and IL-1β often remain unchanged despite evident local inflammation [51,52]. For instance, in rat models of TNBS-induced colitis, plasma cytokine levels measured up to 7 days post-induction were not significantly elevated, despite marked increases in colonic tissue concentrations. Similar results were reported in DNBS models, where elevated colonic mRNA expression of *Tnfa* and *Il1b* was documented, while systemic cytokine expression was not described [53]. These findings support the hypothesis that DNBS-induced colitis in pigs may primarily trigger a local immune response without a strong systemic cytokine reaction. It is also important to consider that ethanol in high doses can have myelotoxic and immunosuppressive effects, leading to a decrease in the number of precursor cells in the bone marrow and impairment of their differentiation [54,55]. Although the ethanol dose was low in our study and administered only once, its local effects may also affect systemic parameters. Furthermore, the possible impact of drugs used for premedication (butorphanol, medetomidine) on hematologic results should also be acknowledged. However, it is worth noting that this scheme and the dosage described are routinely used in studies on pigs conducted in our laboratory. So far, we have not observed deviations in hematological parameters. Moreover, studies by Akbar et al. [56], Samimi et al. [57], Atalan [58], and Sato et al. [59] demonstrated that the use of α2-agonists and opioid drugs (butorphanol) does not significantly affect hematological findings. Nevertheless, the relative contribution of leukocytes, including neutrophils, to the pathogenesis of CD is controversial. While some studies describe their beneficial role, others report their pathological contribution [60,61,62,63]. Their number and role in regulating inflammation strictly depend on the method of colitis induction, the stage of inflammation, and the animal model used.

Calprotectin, which is a calcium-binding protein of the S100 family, is commonly used as a non-invasive marker to assess the degree of intestinal inflammation and disease activity in IBD (active vs. remission) [64]. The results of our study showed that before the administration of DNBS or saline, the fecal calprotectin (FC) concentration did not differ significantly between the two groups. This finding was extremely important for further interpreting the effect of the experimental inflammation model and confirms the homogeneity of the studied group of animals. This result is consistent with the study by Lallès et al. [65], who also found low FC levels in healthy pigs kept in high sanitary conditions. On the other hand, 21 days after DNBS administration, we found a significant, almost 3-fold increase in FC concentration in pigs from the colitis group compared to the control group. Calprotectin, mainly derived from neutrophils and macrophages, is a strong chemotactic factor for neutrophils. During intestinal inflammation, neutrophils are mobilized to migrate to the site of inflammation, causing degranulation and releasing significant amounts of calprotectin into the intestinal lumen [66]. A reduction in the count of #NEU in peripheral blood in our study may also confirm this process. Furthermore, Bjarnason [67] claims that FP concentration provides a non-invasive quantitative measure of neutrophil flow to the intestine. The observed increase in FP levels in the colitis group is consistent with numerous preclinical and clinical studies confirming that calprotectin levels in stool are significantly elevated in patients with IBD, and the degree of elevation is positively correlated with disease severity [68,69,70]. In addition, the study by Amara et al. [71] on DSS-induced colitis in mice also indicates that FC levels correlate with markers of intestinal inflammation, including TNF-α, IL6, and colitis severity. Our results are also consistent with the study by Barbosa et al. [64], who found elevated FC levels in pigs with mucoid or hemorrhagic colitis. The increase in FP revealed in the colitis group confirms that the induction of colitis using DNBS is associated with a marked activation of the inflammatory process in the GIT and that it is a valuable marker in translational IBD research, also due to its high sensitivity and specificity [72].

Plasma liver enzyme activity is commonly used as an indicator of hepatocellular injury and is often elevated in chemically induced colitis models. In our study, ALT activity remained stable across time points and between groups. In contrast, AST activity significantly increased in the colitis group by day 37, compared to the control group and to baseline activities before DNBS administration. These findings are partially consistent with previous studies in TNBS-induced colitis models, where both AST and ALT activities were significantly elevated in plasma, indicating liver function disturbance [73]. The more selective increase in AST observed in our study, without parallel elevation in ALT, may reflect a milder hepatic stress. AST is less liver-specific than ALT and may also originate from muscle [74], although the observed timing and association with colitis induction point toward hepatic origin. The increase in AST may be mediated by gut–liver axis mechanisms, which are well-documented in TNBS and DNBS models. These include intestinal barrier disruption, microbial dysbiosis, and translocation of bacterial products such as lipopolysaccharides (LPS), which can trigger hepatic inflammation [73,75]. Studies have shown that pro-inflammatory cytokines (e.g., TNF-α, IL-1β, IL-6) can be elevated in the liver in such models, contributing to hepatic dysfunction and histological damage. Although our study did not reveal significant systemic elevations of TNF-α or IL-1β, local cytokine production or translocation pathways may still have contributed to hepatic stress. Liver injury is a recognized complication in human IBD. Abnormal liver enzyme activities—particularly elevated AST and ALT—have been reported in up to 30% of IBD patients [76]. Hepatocellular injury, characterized by elevated AST and/or ALT, is one of the more common patterns observed in IBD patients, and it may result from the inflammatory process itself, metabolic dysfunction, or adverse effects of pharmacological treatment [76,77]. Furthermore, the absence of ALT elevation could suggest that the effects of DNBS on the liver are less severe than those reported in TNBS-based models, or that the systemic inflammatory response was not sufficiently robust to cause pronounced liver injury within the study’s timeframe. However, the observed increase in AST highlights the importance of considering the liver function assessment in translational colitis models. It suggests that DNBS-induced colitis in pigs may be suitable for studying such interactions under moderate inflammatory conditions.

Histologic evaluation of the colon, in both CD and UC, is essential to establish a diagnosis and is also valuable in assessing response to therapy [78]. There is also a distinction made for IBD of unspecified type (indeterminate colitis—IC; in cases where there are no clear features to classify the disease into one of the above two entities). UC is characterized by changes that are usually limited to the mucosa in a continuous fashion and to a varying range, extending from the rectum to the more proximal colon [13]. In patients with UC, changes related to intestinal crypts are observed, including the formation of crypt abscesses, crypt architectural distortion (shortening, branching), loss of mucus, metaplasia of Paneth cells, basal lymphoid aggregates, and lamina propria eosinophils. An early symptom indicating UC is also basal plasmocytosis [13]. On the other hand, CD is characterized by involvement of the entire thickness of the intestinal wall (transmural inflammation) and has a multifocal nature (patchy distribution), with an inflammatory cell infiltration in the lamina propria consisting of lymphocytes and plasma cells. A typical feature of CD is also the presence of epithelioid granulomas—such granulomas are visible in 70% of cases of intestinal wall biopsies, but only 15% of cases when the lesions are limited to the mucosa [7]. Our study observed histopathological changes that may be primarily consistent with UC. The most typical feature of the histopathological picture observed in our model was the limitation of changes to the mucosa, which exhibits characteristics typical of UC [13]. Additionally, the inflammatory cell infiltrate was composed of a mixed population of small lymphocytes and plasma cells, which are recognized as changes commonly seen in the early phase of UC (basal plasmocytosis). Mucosal ulcerative changes were observed in 50% of the pigs, with three of these animals having the severity of changes classified as high (3 out of 4 degrees). However, in all cases, the inflammatory infiltrate associated with these lesions did not extend below the submucosal layer, and the inflammatory changes could not be classified as transmural. Such a pattern of change is typical for ulcerative colitis and differs from that observed in CD (in this case, transmural colitis is observed). We also did not see typical inflammatory infiltrates of a granulomatous nature characteristic of CD; therefore, our described model, in histopathological terms, is most similar to what is observed in UC in humans. Furthermore, our morphometric analysis of intestinal crypts objectively revealed a mildly expressed (but statistically significant) shortening of intestinal crypts, which is another morphological feature found in UC [13]. However, in the literature, such changes in crypt histomorphometry are also described as characteristic of CD [79]. Certain morphological differences between our model and UC can be attributed to the fact that our model represents an early stage of the condition. For instance, we observed an absence of colonic crypt abscesses, destruction of goblet cells, and reduced mucus production in our model. These abnormalities, frequently seen in the advanced stages of UC, may not have developed yet in our model.

Erben et al. [80] reported that, among 95 studies on mouse models of IBD, more than half showed inflammation limited to the colon only (65%), while 17% were limited to the small intestine, and 18% involved most or all parts of the colon and small intestine. Clinically, CD affects the small intestine in approximately 30–40% of patients (involving the terminal ileum in 90%), while 40–55% of patients show localization to the ileum and colon, and colonic-only inflammation occurs in only 15–25% of individuals [81]. Therefore, we decided to perform a morphometric analysis of the duodenum, jejunum (proximal, middle, and distal sections), and ileum. Our results showed a significant reduction in the thickness of the muscular layer and the depth of intestinal crypts in the terminal segment of the small intestine—the ileum—in pigs with induced colitis. A reduction in the thickness of the muscle layer may indicate smooth muscle dystrophy, potentially related to chronic inflammatory mediators that can lead to impaired tissue regeneration [82]. In turn, the shortening of crypts may be linked to an imbalance between proliferation and apoptosis of enterocytes. The crypt is a tubular gland formed by the small intestinal epithelium descending into the lamina propria at the base of the villi, and stem cells at their base are responsible for the continuous renewal of the intestinal epithelium [83,84]. Under chronic inflammation conditions, this process is disrupted, resulting in a shortening of the crypts, which often accompanies malabsorption syndrome and malnutrition [85]. Bischoff et al. [28] have reported that increased large intestine permeability may secondarily impair small intestinal barrier function, possibly through weakened tight junctions or endotoxemia. This mechanism may explain the presence of lesions in the ileum, although the main inflammatory stimulus was confined to the large intestine. In addition, it should be mentioned that damage to the small intestine—direct or indirect—can lead to metabolic disorders, which are often observed in patients with IBD. The observed changes in the ileum and segmental inflammation are typical features of CD in humans [8], which are rarely reproduced in rodent models. Due to the similar anatomical length of the gastrointestinal tract and the proportions of the layers of the intestinal wall, the pig model makes it possible to study these features.

The DNBS-induced colitis model in pigs presented in this study shows numerous advantages allowing research on chronic IBD. It successfully reproduces many clinical and histopathological features of this disease. As demonstrated in this study, the model shows high reproducibility and relatively low mortality, making it safe and suitable for long-term experimental IBD protocols. However, as Szkopek et al. [33] rightly point out, there are no perfect experimental animal models, and each model only slightly mimics the clinical condition of the disease entity and requires further improvements and work. As a multifactorial disease, IBD involves interactions between diet, microbiome, environmental factors, genotype, and the intestinal barrier, and no model can holistically capture all these components. Therefore, one of the limiting factors in our study is the lack of a studied microbiome, which is an important aspect to consider in future directions. Another important limiting factor is the short time period (21 days) and single administration of DNBS, which may not capture chronic IBD. It should also be considered that pigs, unlike rodents, have greater space and nutritional requirements, which carry over into higher maintenance costs. Although the DNBS-pig colitis model is not without limitations, its translational value is very high. It allows a complex analysis of the systemic and local inflammatory response under physiologically similar conditions to humans. Given these limitations and the results, we cannot conclusively determine whether the model used is closer to CD or UC, and therefore, it should be treated as an IBD model. However, the model has great potential to contribute to breakthroughs in treating IBD and implementing effective therapies into clinical practice.

## 4. Materials and Methods

### 4.1. Animal Experiment

The experiment was conducted on 16 three-month-old Polish White Landrace male pigs, which were randomly divided into two groups: control and colitis. To avoid the risk of bias, the pigs were divided so that the average body weight with standard deviation in each group was similar. Sample size was estimated using G∗Power software [86], version 3.1.9.4 for a one-way ANOVA at α = 0.05 with 95% power, assuming an effect size (f) = 0.35 for two study groups. The effect size was calculated as the minimum expected mean difference between animals (effect size f = delta Mean/SD). SD was obtained from previously published own studies. Since SDs vary greatly for all the parameters listed, we chose the maximum SD (5.71) for the minimum expected difference in means (2.0). The animals were kept individually in boxes equipped with a water supply and feeder, in which they had constant contact with other animals and received environmental enrichments. The pigs were fed a cereal-based, pelleted, standard diet at 4% of their body weight. The diet contained 19 g/100 g crude protein, 65 g/100 g carbohydrates, 3.9% crude fiber, 3% crude fat, and 6% ash, together with 5000 IU/kg vitamin A, 500 IU/kg vitamin D, and 85 mg/kg vitamin E. The conditions in the housing area were monitored daily and were standard for an experimental pig facility (temperature 21–25 °C, light cycle 12/12, air exchanges 15–20/h, humidity 70 +/− 5%). Body weight in both groups was measured weekly throughout the experiment. Pigs’ weight gains were calculated based on the difference in body weights between the evaluated weeks of the experimental period.

**Figure 7:**
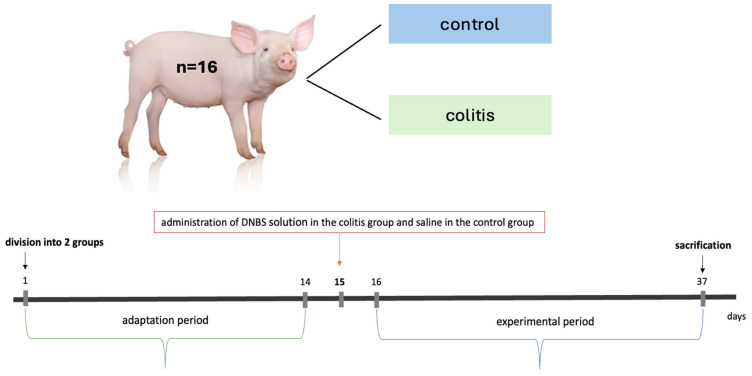
A simple diagram of the study.

After a two-week adaptation period, the animals were premedicated with a combination of medetomidine (1 mg/m^2^; “Domitor”, Orion Pharma, Warsaw, Poland) and butorphanol (0.2 mg/kg body weight; “Torphadine”, Dechra, Warsaw, Poland), intramuscularly. Colitis was induced by a single rectal administration of DNBS (2,4-dinitrobenzenesulfonic acid) dissolved in 50% (*v*/*v*) ethanol at a dose of 80 mg/kg body weight. The concentration of DNBS was approximately 8.22% *v*/*v*. The final density of the ethanolic DNBS solution was approximately 0.977 g/cm^3^. The DNBS dose was 80 mg/kg body weight, which comes out to 1 mL of ethanolic DNBS solution per kilogram of body weight. The administration was via an intragastric probe made of medical PVC (polyvinyl chloride) with dimensions of 10.6 mm × 76 cm (Sigmed, Cisek, Poland; cat. no. 91951) coated with lignocaine gel under ultrasound control into the descending colon. After administration of DNBS, the animals were kept on the surgical table at an angle of 30° for 30 min. Anesthesia was maintained with inhaled isoflurane (1–1.5%, oxygen flow 2–2.5 L/minute; “Isotek”, Vet-Agro, Lublin, Poland). Animals in the control group were administered a 0.9% NaCl solution in the same volume and way. Then, a 21-day observation period was initiated. A simple diagram of the study design is shown in Figure 7.

The experimental procedures for this study were approved by the II Local Ethics Committee for Animal Experiments in Warsaw, Poland (Resolution No. WAW2/090/2023). All procedures involving animals were performed in accordance with the Polish Law for the Care and Use of Animals, EU regulations (Directive 2010/63/EU), and the Code of Ethics of the World Medical Association (Declaration of Helsinki). The present experiment also complied with the ARRIVE guidelines 2.0 [87].

#### 4.1.1. Evaluation of the Clinical Condition of Animals

For 21 days, the clinical conditions of the animals in both groups were monitored daily after administration of DNBS or 0.9% NaCl. The abdominal pain (animal behavior and palpation examination) and quality and frequency of feces were evaluated on a scoring scale. The scoring scale is shown in Table 6 and Supplementary Table S2. Additional clinical parameters were assessed, including appetite (present, decreased, absent), thirst (present, decreased, absent), locomotor activity (normal, decreased, absent), internal temperature (normal, increased, decreased), number of breaths per minute (normal, increased, decreased), interaction with the environment (normal, decreased or absent), and degree and % dehydration (palpation and total protein with hematocrit test) (Supplementary Table S3).

#### 4.1.2. Sampling Procedure and Autopsy

On days 15 (before DNBS administration) and 37 (end of experiment), blood was collected from the animals’ external jugular vein into EDTA tubes (BD Vacutainer, Becton Dickinson, NJ, USA) for morphological and biochemical analysis. In addition, on the same days, feces samples were collected to determine calprotectin concentration.

At the end of the study, all pigs were sacrificed by a single dose of intravenously injected sodium pentobarbiturate (140 mg/kg body weight; “Morbital”, Biowet, Pulawy, Poland), after prior intramuscular premedication with a mixture of medetomidine (0.1 mg/kg body weight; “Domitor”, Orion Pharma, Warsaw, Poland) and butorphanol (0.2 mg/kg body weight; “Torphadine”, Dechra, Warsaw, Poland). Subsequently, samples from the small intestine were taken for histomorphometry analysis, as well as a section of the colon for histopathological examination and histomorphometry analysis.

### 4.2. Hematological and Biochemical Examination of the Blood

Blood testing was performed on days 15 and 37 of the experiment using a 5-DIFF Mythic 5VET PRO automatic veterinary hematology analyzer (Cormay, Warsaw, Poland). The following parameters were evaluated: white blood cells count (WBC), percentage and absolute counts of lymphocytes (LYM%, LYM#), monocytes (MON%, MON#), of neutrophils (NEU%, NEU#), eosinophils (EOS%, EOS#), basophils (BASO%, BASO#), red blood cell count (RBC), hemoglobin concentration (HGB), hematocrit (HCT), mean corpuscular volume (MCV), mean corpuscular hemoglobin (MCH), mean corpuscular hemoglobin concentration (MCHC), red blood cell distribution width-coefficient of variation (RDW_CV) and platelet count (PLT). Blood plasma was obtained by centrifugation at 3000× *g* for 10 min at 4 °C. The resulting plasma samples were subsequently stored at −80 °C until biochemical analyses were performed. Plasma enzyme activities of alanine (ALT) and aspartate (AST) aminotransferases were measured using kinetic assays with commercially available kits (Sigma Aldrich, Saint Louis, MO, USA).

### 4.3. Assessment of Plasma Cytokine Levels

Tumor necrosis factor-alpha (TNF-α) and interleukin-1 beta (IL-1β) concentrations in plasma were measured using specific quantitative sandwich ELISA kits (Porcine TNF-alpha ELISA Kit (cat. No. RAB0478), Porcine IL-1 beta/IL-1F2 ELISA Kit (cat. No. RAB0276) (Sigma Aldrich, Saint Louis, MO, USA) according to the manufacturer’s instructions.

### 4.4. Examine Fecal Calprotectin Levels

Fecal calprotectin levels on days 15 and 37 were quantified using a commercially available ELISA test (MyBioSource, San Diego, CA, USA; Cat. No. MBS2801875), according to the manufacturer’s instructions. Briefly, stool samples were homogenized in the provided extraction buffer to achieve a consistent suspension and subsequently centrifuged to remove particulate matter. The resulting supernatants were transferred to 96-well microplates pre-coated with a monoclonal antibody specific to calprotectin. After incubation and washing, a biotinylated detection antibody was added, followed by HRP-conjugated streptavidin. The enzymatic reaction was developed using a TMB substrate and terminated with a stop solution. Optical density was measured at 450 nm using a microplate reader (Multikan Sky, Thermo Scientific, Rockford, IL, USA). Calprotectin concentrations were calculated based on a standard curve generated using known concentrations of recombinant porcine calprotectin provided in the kit.

### 4.5. Histopathological Evaluation of Porcine Colon and Small Intestine

The colon sections and samples from the small intestine were prepared and stained for routine histopathological and histomorphometric analysis [88]. Briefly, collected macroscopically altered colon specimens, as well as samples from the duodenum, proximal, middle, and distal parts of the jejunum, and the ileum were fixed in 10% neutral buffered formalin for at least 24 h, then processed via standard protocols (dehydration, clearing, and paraffinization). Sections from a paraffin block with colon were cut at 4 µm thickness, while sections from the small intestine were cut at 4.5 µm thickness, and stained with hematoxylin–eosin. Histopathological analysis of the colon was performed according to the Geboes scoring scale (Table 7) [30], which evaluates the composition and intensity of the inflammatory cell infiltrate, crypts destruction, and the presence of erosions and ulcers.

**Figure 8:**
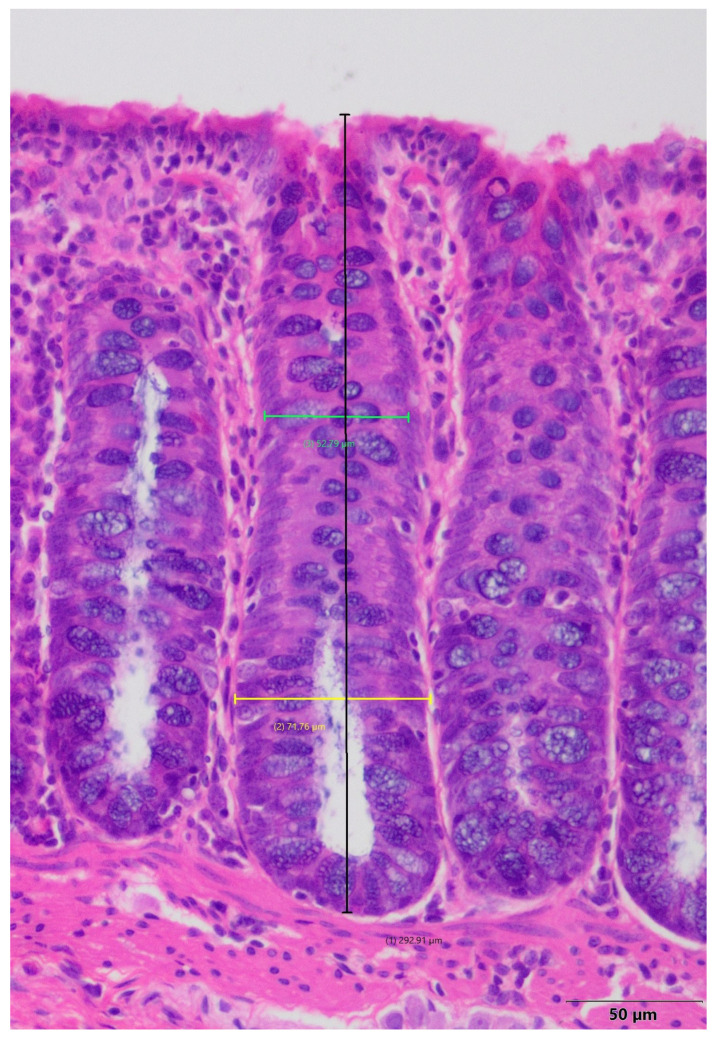
Microscopic view of colonic mucosa presenting colonic crypt with measurement points: black line—length of the crypt, yellow line—width of the lower part, green line—width of the upper part; hematoxylin–eosin stain, magnification 200×.

A histomorphometric examination of colonic crypts, including their length and width, was performed to provide a more detailed characterization of inflammation-induced changes in the colon. The samples selected for histomorphometry were those of the highest quality. The histomorphometric analyses were conducted using an Olympus BX41 microscope (Olympus, Tokyo, Japan) coupled to a computer equipped with the CellA^®^ analysis system. For each colon specimen, areas with clearly visible and well-stained crypts with visible histologic elements were selected. In each sample, 30 crypts were analyzed. The software was used to measure the length of the crypt and its width in the upper and lower parts (Figure 8).

In the histomorphometric analysis of the small intestine, parameters such as the thickness of the muscular lamina and mucosa, the length of the villi, and the depth of the crypts were measured [88]. Images were analyzed using a light microscope (Axioskop 40, Zeiss, Jena, Germany) with a digital camera (Coolpix B700, Nikon, Tokyo, Japan). The obtained data were accessed using Axio Vision software version 4.2 (Zeiss, Germany). A minimum of 20 measurements of each parameter were made for each section.

### 4.6. Statistical Analysis

The data obtained from hematological analyses were analyzed using Statistica software (version 13.3 EN; StatSoft, Krakow, Poland). Before conducting further analyses, the equality of variance and normality of distribution were determined for all datasets. To enable statistical analysis, the following data were transformed to obtain the normal distribution and equal variance by the square root (length and width of the upper part of the intestinal crypts). Based on Cook’s distance analysis, individual values in some parameters were excluded from the statistical analysis. The unpaired *t*-test was used to indicate the statistical differences between the groups, or the Mann–Whitney test was used to determine if the assumptions of equality of variance and normal distribution were unmet. Statistical significance was established at a *p*-value of less than 0.05.

## 5. Summary and Conclusions

**Figure 9:**
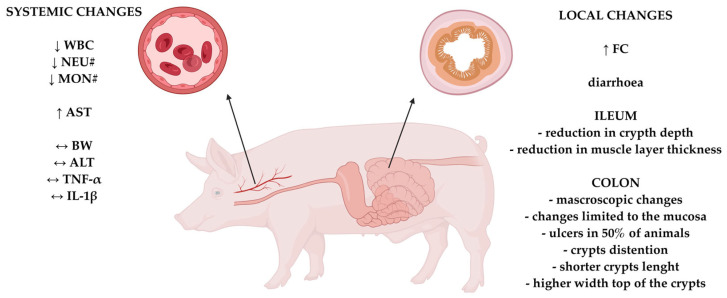
Diagram summarizing systemic and local changes caused by DNBS-induced local inflammation in the colon. Abbreviation: FC—fecal calprotectin concentration; WBC—white blood cells count; NEU#—neutrophil count; MON#—monocyte count; AST—aspartate aminotransferase activity in blood, BW—body weight; ALT—alanine aminotransferase activity in blood; TNF-α—tumor necrosis factor alpha; IL-1β—interleukin 1 beta. ↓—decrease; ↑—increase; ↔—no change.

This study demonstrates that rectal administration of DNBS (in 50% (*v*/*v*) ethanol at a dose of 80 mg/kg body weight) in domestic pigs induces colonic inflammation with clinical, biochemical, and histopathological features resembling human IBD. The model reproduced key characteristics of IBD—including diarrhea, mucosal ulceration, crypt architecture distortion, and elevated fecal calprotectin—while maintaining good general condition and low mortality, supporting its feasibility for long-term studies (Figure 9). Histologically, the inflammation was primarily limited to the mucosa and submucosa, mimicking ulcerative colitis. However, segmental involvement of the ileum and alterations in crypt depth and muscularis thickness suggest some overlap with Crohn’s disease pathology. Compared to rodent models, the pig model offers greater anatomical and physiological similarity to humans. These findings highlight the translational potential of the DNBS-induced colitis pig model for studying IBD pathophysiology and for preclinical testing of novel therapies, including dietary, probiotic, and pharmacological interventions. These results indicate that we have successfully developed a technically feasible and minimally invasive model of colitis in pigs, which better preserves animal welfare than other available methods. This model can be effectively used to study the pathophysiology of IBD and for preclinical testing of novel therapeutic approaches, including dietary, probiotic, and pharmacological interventions. However, further studies are needed to explore the model’s kinetics over a longer experimental period and/or to assess the effects of multiple doses of DNBS to induce chronic colitis. Such investigations would provide a more comprehensive understanding of the model and allow a better evaluation of its similarity to Crohn’s disease or ulcerative colitis.

## Figures and Tables

**Table 1:** Scoring of abdominal pain, fecal quality, and frequency of animals.

Control Group																
Pig Number	1		2		3		4		5		6		7		8	
Days After Administration of NaCl	AP	FQF	AP	FQF	AP	FQF	AP	FQF	AP	FQF	AP	FQF	AP	FQF	AP	FQF
1	0	0	0	0	0	0	0	0	0	2	0	1	0	2	0	2
2	0	0	0	0	0	0	0	0	0	2	0	0	0	0	0	2
3	0	0	0	0	0	0	0	2	0	2	0	0	0	0	0	0
4	0	0	0	0	0	0	0	2	0	2	0	0	0	1	0	1
5	0	0	0	0	0	2	0	2	0	1	0	0	0	0	0	0
6	0	0	0	0	0	1	0	1	0	1	0	0	0	0	0	0
7	0	0	0	0	0	1	0	1	0	1	0	0	0	0	0	0
8	0	0	0	0	0	0	0	0	0	1	0	0	0	1	0	0
9	0	0	0	0	0	1	0	1	0	1	0	0	0	0	0	0
10	0	0	0	0	0	1	0	0	0	1	0	0	0	1	0	0
11	0	0	0	0	0	0	0	0	0	0	0	0	0	0	0	0
12	0	0	0	0	0	0	0	1	0	0	0	0	0	0	0	0
13	0	0	0	0	0	0	0	1	0	0	0	0	0	0	0	0
14	0	0	0	0	0	0	0	1	0	0	0	1	0	0	0	0
15	0	0	0	0	0	0	0	1	0	1	0	0	0	0	0	0
16	0	0	0	0	0	0	0	0	0	0	0	0	0	0	0	0
17	0	0	0	0	0	0	0	0	0	0	0	0	0	0	0	0
18	0	0	0	0	0	0	0	0	0	0	0	0	0	0	0	0
19	0	0	0	0	0	0	0	0	0	0	0	0	0	0	0	0
20	0	0	0	0	0	0	0	0	0	1	0	0	0	0	0	0
21	0	0	0	0	0	0	0	0	0	0	0	0	0	0	0	0
**Colitis Group**																
**Pig Number**	**9**		**10**		**11**		**12**		**13**		**14**		**15**		**16**	
**Days After Administration of DNBS**	**AP**	**FQF**	**AP**	**FQF**	**AP**	**FQF**	**AP**	**FQF**	**AP**	**FQF**	**AP**	**FQF**	**AP**	**FQF**	**AP**	**FQF**
1	0	2	1	0	0	2	0	2	0	2	0	3	0	2	1	0
2	0	2	0	0	0	2	0	2	0	2	0	2	0	0	0	2
3	0	2	0	0	0	3	0	3	0	2	0	2	0	2	0	2
4	0	2	0	2	0	0	0	2	0	2	0	2	0	2	0	2
5	0	2	0	2	0	2	0	3	0	2	0	1	0	2	0	2
6	0	3	0	2	0	2	0	3	0	2	0	0	0	2	0	2
7	0	2	0	2	0	2	0	2	0	2	0	0	0	2	0	0
8	0	2	0	0	0	0	0	2	0	2	0	0	0	2	0	0
9	0	2	0	1	0	0	0	2	0	2	0	0	0	0	0	0
10	0	2	0	2	0	0	0	2	0	2	0	0	0	2	0	0
11	0	2	0	0	0	0	0	1	0	2	0	2	0	2	0	0
12	0	2	0	0	0	2	0	2	0	2	0	1	0	2	0	0
13	0	2	0	2	0	0	0	1	0	2	0	1	0	2	0	0
14	0	2	0	2	0	1	0	2	0	2	0	1	0	2	0	0
15	0	2	0	0	0	2	0	1	0	2	0	2	0	0	0	0
16	0	2	0	1	0	2	0	1	0	2	0	1	0	0	0	1
17	0	2	0	1	0	1	0	1	0	2	0	1	0	0	0	0
18	0	0	0	1	0	0	0	2	0	2	0	1	0	0	0	1
19	0	0	0	1	0	2	0	2	0	2	0	0	0	0	0	1
20	0	1	0	2	0	2	0	2	0	2	0	0	0	0	0	1
21	0	1	0	2	0	1	0	2	0	2	0	0	0	0	0	0

Abbreviations: AP—abdominal pain, FQF—fecal quality and frequency. The clinical condition in the control group (saline administration) and the colitis group (DNBS administration) was monitored for 21 days after administration. AP: 0—no abdominal pain, 1—moderate abdominal pain, 2—severe abdominal pain. FQF: 0—feces properly formed, without pathological additions, frequency normal, 1—feces properly formed, but too soft, or feces partially abnormally formed, without pathological additions, frequency normal, 2—diarrhea, without pathological additions, normal or increased frequency, 3—diarrhea with blood or with a lot of mucus, increased frequency.

**Table 2:** The plasma activities of liver enzymes.

Feature	Experimental Group				Statistical Significance
	Control 15 d	Colitis 15 d	Control 37 d	Colitis 37 d	
AST [mU/mL]	35.98 ± 4.54	37.19 ± 4.42 ##	38.32 ± 3.56	54.75 ± 4.79 **	** *p* = 0.0153
ALT [mU/mL]	35.55 ± 3.80	32.92 ± 3.40	56.11 ± 10.28	65.44 ± 10.33	NS

The data are presented as mean ± SEM, *n* = 8. Significant differences are indicated by ** (*p* < 0.01) vs. the control group at the same time point, and ## (*p* < 0.01) between experimental time points within the colitis group.

**Table 3:** The plasma levels of cytokines.

Feature	Experimental Group				Statistical Significance
	Control 15 d	Colitis 15 d	Control 37 d	Colitis 37 d	
TNF-α [pg/mL]	29.93 ± 9.44	25.49 ± 7.50	23.75 ± 5.15	20.65 ± 4.40	NS
IL-1β [pg/mL]	85.54 ± 23.67	81.08 ± 25.17	68.35 ± 16.50	54.25 ± 10.11	NS

The data are presented as mean ± SEM, *n* = 8. Abbreviations: TNF-α—tumor necrosis factor-alpha; IL-1β—interleukin-1 beta.

**Table 4:** Histopathological evaluation of the colon.

Pig Number	General Architecture	Chronic Inflammatory Infiltrate	Lamina Propria Neutrophils	Lamina Propria Eosinophils	Neutrophils in Epithelium	Crypt Destruction	Erosion and Ulceration
**Control Group**							
1	0	1	0	1	1	0	0
2	0	1	0	2	1	0	0
3	0	1	0	1	1	0	0
4	1 crypt distension	1	0	0	0	0	0
5	0	1	1	1	0	0	0
6	0	1	0	1	0	0	0
7	0	1	0	0	0	0	0
8	0	1	0	0	0	0	0
**Colitis Group**							
9	1 crypt distension	1	0	2	1	0	0
10	1 crypt distension	2	0	2	1	0	1
11	1 crypt distension	2	1	1	1	0	3
12	1 crypt distension	2	1	1	1	0	3
13	1 crypt distension	2	1	1	1	0	3
14	0	1	0	1	0	0	0
15	0	2	0	1	0	0	0
16	0	1	0	1	0	0	0

Histopathological evaluation based on the Geboes scale. General architecture: 0—no abnormality, 1—mild abnormality, 2—mild or moderate diffuse or multifocal abnormalities, 3—severe diffuse or multifocal abnormalities; chronic inflammatory infiltrate: 0—no increase, 1—mild but unequivocal increase, 2—moderate increase, 3—marked increase; lamina propria neutrophils: 0—none, 1—mild but unequivocal increase, 2—moderate increase, 3—marked increase; lamina propria eosinophils: 0—none, 1—mild but unequivocal increase, 2—moderate increase, 3—marked increase; neutrophils in epithelium: 0—none, 1—<5% crypts involved, 2—<50% crypts involved, 3—>50% crypts involved; erosion or ulceration: 1—no erosion, ulceration, or granulation tissue, 1—recovering epithelium + adjacent inflammation, 2—probable erosion (focally stripped), 3—ulcer or granulation tissue.

**Table 5:** Histomorphometric analysis of the small intestine.

Parameter/Group	Control Group	Colitis Group
	Duodenum	
Villus length [μm]	1128.11 ± 10.92	1104.64 ± 12.60
Mucosa thickness [μm]	935.19 ± 4.52	910.66 ± 14.24
Crypt depth [μm]	320.26 ± 3.46	311.73 ± 4.00
Muscularis thickness [μm]	653.15 ± 3.71	642.64 ± 8.72
	Proximal Jejunum	
Villus length [μm]	984.01 ± 6.32	993.55 ± 15.92
Mucosa thickness [μm]	914.42 ± 5.70	903.10 ± 10.75
Crypt depth [μm]	254.78 ± 2.48	252.70 ± 2.83
Muscularis thickness [μm]	594.75 ± 7.55	577.00 ± 12.66
	Middle Jejunum	
Villus length [μm]	949.42 ± 6.59	941.34 ± 16.94
Mucosa thickness [μm]	842.68 ± 9.78	831.30 ± 7.53
Crypt depth [μm]	250.91 ± 1.69	250.15 ± 3.53
Muscularis thickness [μm]	568.00 ± 5.48 ^a^	559.31 ± 9.70 ^b^
	Distal Jejunum	
Villus length [μm]	916.30 ± 7.19	907.15 ± 9.37
Mucosa thickness [μm]	812.72 ± 6.88	805.45 ± 12.62
Crypt depth [μm]	249.25 ± 2.29	246.12 ± 3.24
Muscularis thickness [μm]	534.41 ± 3.89	530.36 ± 5.19
	Ileum	
Villus length [μm]	864.32 ± 7.64	851.66 ± 12.93
Mucosa thickness [μm]	760.08 ± 6.56	738.85 ± 10.23
Crypt depth [μm]	236.38 ± 2.14 ^a^	229.16 ± 2.30 ^b^
Muscularis thickness [μm]	521.55 ± 3.68 ^a^	503.71 ± 6.29 ^b^

Results are presented as the mean ± SEM. Significant differences are indicated by different letters (*p* < 0.05).

**Table 6:** Animal condition point scale.

Abdominal Pain	
Points	Description
0	No abdominal pain
1	Moderate abdominal pain
2	Severe abdominal pain
	Fecal quality and frequency
0	Feces properly formed, without pathological additions, frequency normal
1	Feces properly formed, but too soft, or feces partially abnormally formed, without pathological additions, frequency normal
2	Diarrhea, without pathological additions, normal or increased frequency
3	Diarrhea with blood or with a lot of mucus, increased frequency

**Table 7:** Geboes scoring scale.

General Architecture	
Points	Description
0	No abnormality
1	Mild abnormality
2	Mild or moderate diffuse or multifocal abnormalities
3	Severe diffuse or multifocal abnormalities
	Chronic inflammatory infiltrate
0	No increase
1	Mild but unequivocal increase
2	Moderate increase
3	Marked increase
	Lamina propria neutrophils
0	None
1	Mild but unequivocal increase
2	Moderate increase
3	Marked increase
	Lamina propria eosinophils
0	None
1	Mild but unequivocal increase
2	Moderate increase
3	Marked increase
	Epithelium neutrophils
0	None
1	<5% crypts involved
2	<50% crypts involved
3	>50% crypts involved
	Crypt destruction
0	None
1	Probable—local excess of neutrophils in part of crypt
2	Probable—marked attenuation
3	Unequivocal crypt destruction
	Erosion or ulceration
0	No erosion, ulceration, or granulation tissue
1	Recovering epithelium + adjacent inflammation
2	Probable erosion—focally stripped
3	Unequivocal erosion
4	Ulcer or granulation tissue

## Data Availability

The original data presented in the study are openly available in the Repository for Open Data (RepOD) at https://doi.org/10.18150/RGKXLF.

## Supplementary Materials

The following supporting information can be downloaded at: https://www.mdpi.com/article/10.3390/ijms26189115/s1.

## Abbreviations

IBD	Chronic inflammatory bowel disease
CD	Crohn’s disease
UC	Ulcerative colitis
GIT	Gastrointestinal tract
DNBS	2,4-dinitrobenzenesulfonic acid
TNBS	2,4,6-trinitrobenzene sulfonic acid
DSS	Dextran sulfate sodium
BW	Body weight
BWG	Body weight gain
AP	Abdominal pain
FQF	Fecal quality and frequency
WBC	White blood cells
NEU#	Absolute value of neutrophils
MON#	Absolute value of monocytes
IETs	Intraepithelial layer
FC	Fecal calprotectin
TNF-α	Tumor necrosis factor-alpha
IL-1β	Interleukin-1 beta
ALT	Alanine aminotransferase
AST	Aspartate aminotransferase
