# Supplementary material for: Efficacy of 2,4-Dinitrobenzenesulfonic Acid (DNBS) in the Maintenance of a Model of Inflammatory Bowel Disease in Pigs (Sus scrofa domestica)
Source: Int J Mol Sci. 2025 Sep 18;26(18):9115. doi: 10.3390/ijms26189115 (PMC12470971; doi:10.3390/ijms26189115)
Supplement: Supplementary file 1 [file ijms-26-09115-s001.zip › Supplementary Table S2_Modified Unesp-Botucatu pain scale.pdf]

In each category, the animal could receive a maximum of 2 points, where 0 corresponded to physiology and 2 to visible pathology. The points from the scale were then added up, and if the score was ≥4 points, the animal received pain medication and was excluded from the study.

Modified Unesp-Botucatu pain scale

Interaction	Active, observes the environment, interacts/follows other animals	0
	Apathetic, may remain near other animals but interactions are reduced	1
	Very apathetic: isolated and does not interact with other animals, not interested in the environment	2
Mobility	Moves freely, movement is normal, when stopping, the front limbs remain parallel to the hind limbs	0
	Moves with restriction, shortened stride, pauses or limping; when stopped, the front limbs are turned outwards and further back than normal	1
	Difficulty and reluctance to get up, does not move or walks with a limp, may lean against walls	2
Head position	Head above the neck	0
	Head at neck level	1
	Head below the neck (except when eating)	2
Posture	Rounded back, extends head and neck, lies with head on or close to the ground, rapid repeated tail movements or raises tail (except during defecation/urination)	
	None of the above	0
	One of the above	1
	Two or more of the above	2
Activity	Moves normally	0
	Restless, moves more than normal or lies down and gets up frequently	1
	Moves only when encouraged by humans or does not move at all	2
Appetite	Normorexia	0
	Hyporexia	1
	Anorexia	2
Abdominal palpation	No response, animal calm, not interested in the abdominal area	0
	Anxiety, looks nervously or closes eyes, grinds one’s teeth	1
	Very anxious, runs away, does not allow itself to be touched in the abdominal area	2
