# Supplementary material for: Efficacy of 2,4-Dinitrobenzenesulfonic Acid (DNBS) in the Maintenance of a Model of Inflammatory Bowel Disease in Pigs (Sus scrofa domestica)
Source: Int J Mol Sci. 2025 Sep 18;26(18):9115. doi: 10.3390/ijms26189115 (PMC12470971; doi:10.3390/ijms26189115)
Supplement: Supplementary file 1 [file ijms-26-09115-s001.zip › Supplementary Table S3_Clinical condition.pdf]

The animals were observed systematically daily. The table below presents the classified symptoms that may occur in experimental animals. Depending on the severity of the symptoms, treatment and further monitoring of the animals will be undertaken, or they will be excluded from the study, or a decision will be made to euthanize them humanely. Individual points from successive categories were added up and, depending on the total number of points, a decision was made regarding further treatment.

Procedure:
Below 3 points - physiological condition unchanged.
4-5 points - close monitoring of the patient, initial treatment.
6-9 points - correction of the treatment plan, close monitoring of the patient, exclusion from the study.
10 and above - humane euthanasia of the animal.

Category	Animal condition	Score
Food intake	Normal	0
	No appetite	1
Water intake	Normal	0
	None	1
Physical activity	Normal	0
	Patient immobile (no typical physical activity)	5
	Limb fracture	10
Body temperature	Normal	0
	Hypothermia/hyperthermia	5
Breathing	Normal	0
	Increased/decreased respiratory rate	1
Red blood cel count	Normal	0
	Anemia/polycythemia	5
Albumin	Normal	0
	Decrease/increase	1
Total protein	Normal	0
	Decrease/increase	1
Globulin	Normal	0
	Decrease	1
Diarrhea	Feces properly formed, without pathological additions, frequency normal	0
	Feces properly formed, but too soft, or feces partially abnormally formed, without pathological additions, frequency normal	1
	Diarrhea, without pathological additions, normal or increased frequency	2
	Diarrhea with blood or with a lot of mucus, increased frequency	3
Body weight	25% weight loss	5
	50% weight loss	10
Dehydration	Less than 5%	0
	10-12%	5
	13-15%	10
Abdominal pain	None	0
	Moderate	1
	Severe	2
