# Supplementary figures and images for: Efficacy of 2,4-Dinitrobenzenesulfonic Acid (DNBS) in the Maintenance of a Model of Inflammatory Bowel Disease in Pigs (Sus scrofa domestica)
Source: Int J Mol Sci. 2025 Sep 18;26(18):9115. doi: 10.3390/ijms26189115 (PMC12470971; doi:10.3390/ijms26189115)

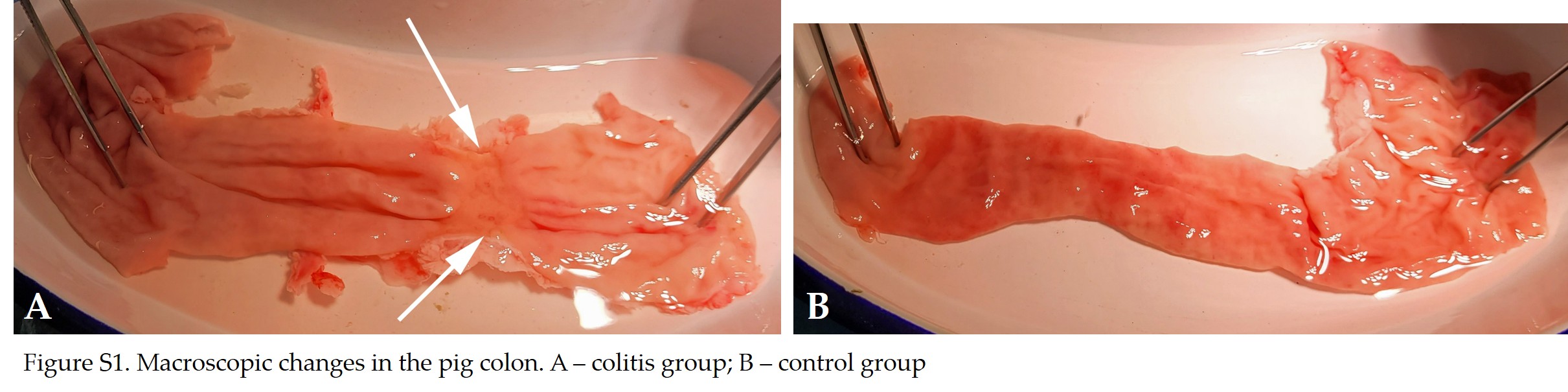

Supplement: Supplementary file 1 [file ijms-26-09115-s001.zip › Supplementary Figure S1_Macroscopic changes in the colon.jpg]
